# Mutations of Pre-mRNA Splicing Regulatory Elements: Are Predictions Moving Forward to Clinical Diagnostics?

**DOI:** 10.3390/ijms18081668

**Published:** 2017-07-31

**Authors:** Lucie Grodecká, Emanuele Buratti, Tomáš Freiberger

**Affiliations:** 1 Centre for Cardiovascular Surgery and Transplantation, Brno 65691, Czech Republic; lucie.grodecka@cktch.cz; 2 International Centre for Genetic Engineering and Biotechnology, 34149 Trieste, Italy; buratti@icgeb.org; 3 Central European Institute of Technology, Masaryk University, Brno 62500, Czech Republic

**Keywords:** splicing regulatory elements, in silico predictions, pre-mRNA splicing, mutation, evaluation of prediction tools, variants of unknown significance, splicing aberration

## Abstract

For more than three decades, researchers have known that consensus splice sites alone are not sufficient regulatory elements to provide complex splicing regulation. Other regulators, so-called splicing regulatory elements (SREs) are needed. Most importantly, their sequence variants often underlie the development of various human disorders. However, due to their variable location and high degeneracy, these regulatory sequences are also very difficult to recognize and predict. Many different approaches aiming to identify SREs have been tried, often leading to the development of in silico prediction tools. While these tools were initially expected to be helpful to identify splicing-affecting mutations in genetic diagnostics, we are still quite far from meeting this goal. In fact, most of these tools are not able to accurately discern the SRE-affecting pathological variants from those not affecting splicing. Nonetheless, several recent evaluations have given appealing results (namely for EX-SKIP, ESRseq and Hexplorer predictors). In this review, we aim to summarize the history of the different approaches to SRE prediction, and provide additional validation of these tools based on patients’ clinical data. Finally, we evaluate their usefulness for diagnostic settings and discuss the challenges that have yet to be met.

## 1. Introduction

One of the most important features distinguishing prokaryotic from eukaryotic gene expression is the process of RNA splicing. During splicing, the borders of intervening sequences (so-called introns) are recognized, cleaved, and exons are then ligated together. This process is catalyzed by a large ribonucleoprotein complex termed spliceosome. Through multiple protein–RNA and RNA–RNA interactions, this huge molecular machine recognizes pre-mRNA sequence elements indicated as “splicing signals”. These include: donor splice site, branch point site and acceptor splice site. Importantly, mammalian genomes contain a huge number of pseudo splice sites, i.e. sequences that resemble real splice sites but are never used under normal conditions. These pseudo sites outnumber the authentic splice sites by an order of magnitude. Therefore, additional regulatory sequences are necessary to discern between the two and are referred to as “splicing regulatory elements” (SREs). The function of the SREs is to bind splicing activators or repressors and influence the choice of adjacent splice sites. In general, SREs play a key role both in constitutive splicing as well as the regulation of alternative splicing (the process through which more than one mRNA isoform can be created from a single precursor transcript) [1,2,3].

Compared to the relatively conserved splice sites, the SRE sequences are much more degenerate [4]. SREs were originally categorized according to their localization and effect on splicing as exonic or intronic splicing enhancers (ESEs and ISEs) and silencers (ESSs and ISSs). Despite still being in use, this division now seems to be rather simplistic, as some SREs can even act adversely from various exonic or various intronic positions (e.g., SRp40 binding elements act mostly as silencers in *ADAR2* exon 8, but behave as enhancers in about 25% of positions when shifted within the same exon; similarly, neuron-specific splicing factors Nova activate alternative exon inclusion when bound to the downstream intron, but repressed its inclusion when bound to the upstream intron) [2,5]. Importantly, most regulatory contexts emerge from overlapping RNA elements. The existence of composite exonic regulatory elements of splicing (CERES) proves that some of the overlapping elements may have both silencing and enhancing properties [6]. Complicating the matter a little bit more, the elements’ recognition is also influenced by its accessibility, i.e. the state of chromatin and RNA secondary structure [2].

In addition, SREs can influence not only the standard pre-mRNA splicing process but most probably also backsplicing—a special type of alternative splicing which leads to circular RNA (circRNA) formation. circRNAs are abundant, stable and evolutionary conserved noncoding RNAs often expressed in a tissue specific manner. The tissue specificity and the lack of correlation between expression levels of a circRNA and the linear transcript from which it is derived indicate that the process of circRNA biogenesis may be precisely regulated [7]. Supporting the role of SREs in the circRNA biogenesis, several splicing regulators have been found to be implicated in this process. In particular, regulation of circRNAs formation was demonstrated for splicing factor Muscleblind and several hnRNP and SR family proteins in Drosophila [8,9]. Similarly, splicing regulators QKI, RBM20 and FUS have been described to activate or repress biogenesis of specific circRNAs in human [10,11,12].

In theory, the mutation of any *cis*-acting splicing element may result in aberrant splicing. In accordance, pre-mRNA splicing defects are responsible for a substantial proportion of inherited disorders (estimated between 15% and 50%) [4]. Notably, since deregulation of specific circRNAs levels has already been shown to be associated with human diseases, this could be another way through which SRE sequence variants might lead to pathology [7]. However, further research will be needed to confirm the role of particular SRE aberrations in circRNAs genesis and disease development.

Although some exons seem to be especially prone to SRE aberrations [13,14,15], several systematic studies showed that SRE changes generally lead to splicing defects much less frequently than splice site disruptions [16,17,18,19]. Still, SRE-affecting mutations impose a significant burden on genetic diagnostics, as they can occur virtually anywhere in the exons or introns and are extremely difficult to be distinguished from harmless changes, which are also very abundant in human genomes [20]. This issue is even more pronounced now, as the next generation sequencing produces thousands of novel variants with every single read [21].

For all these reasons, many potential SRE-affecting variants fall into the category of so-called “variants of unknown significance” [20]. To distinguish these mutations from harmless non splicing-affecting variants, medical geneticists can either use laborious in vitro studies or much more feasible (yet less reliable) in silico predictions [22]. In this review, we summarize the current state of SRE predictions and evaluate their reliability and potential use in clinical diagnostic settings.

## 2. Predictions on SREs

**Figure 1:**
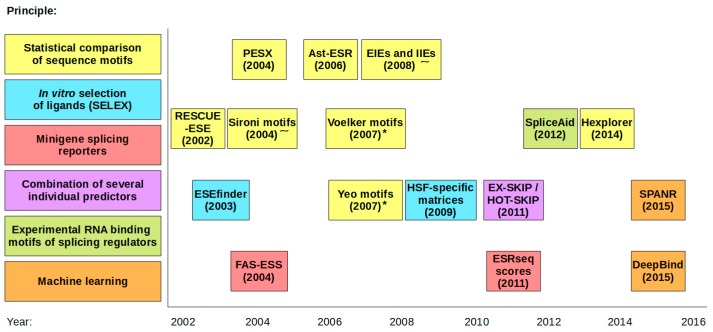
Individual approaches to SRE predictions showing the first release of the online tools (* denotes the tools that are accessible via Sroogle engine; ˜ denotes the tools that are accessible via Human Splicing Finder (HSF) engine).

Following the striking finding that virtually any DNA change may affect splicing by disrupting potential SREs, many attempts to locate these elements have been performed. Some systematic approaches then led to the development of several SRE-predicting tools that are listed in Table 1 and Figure 1. At first, Liu et al. attempted to define ESE motifs by using the method of “functional systematic evolution of ligands by exponential enrichment” (functional SELEX). They used this in vitro selection of splicing activating sequences to determine the binding preferences of four classical SR-proteins, SRSF1, SRSF2, SRSF5 and SRSF6 [23]. Based on these matrices, Cartegni et al. then developed an online tool called ESE-finder [24]. The major advantage of this approach is that users can easily link any particular splicing-affecting sequence with its cognate binding factor. On the other hand, all sequence variants often induce ambiguous changes in the predicted scores for several individual factors, so the conclusions about the variant’s effect on splicing may not be very straightforward. In addition, there is no clue what score changes are sufficient to impact splicing events, because threshold values indicated in the program may be specific for the used experimental conditions [23]. The same approach (SELEX) was later used to assign binding preferences to other splicing factors, such as hnRNPA1, Tra2beta, 9G8 and U2AF [25,26].

Next, Fairbrother et al. presented another pioneering approach to identifying ESEs: Relative Enhancer and Silencer Classification by Unanimous Enrichment (RESCUE) [39]. They compared the occurrence of all possible hexanucleotide motifs in exonic and intronic sequences and both in exons with strong and weak splice sites. The rationale behind such an experiment is that ESEs are more prevalent in exons than in introns and that exons with weak splice sites are more dependent on the presence of ESEs. The authors made their results easily accessible with an online tool named RESCUE-ESE [36]. This tool is easy to use, simply showing the number and identity of predicted ESEs. However, there is no correction for possible elements’ overlap and for any related context dependence.

Other scientists later adapted the same principle for further SRE identification, only using different rationales. From those, we would like to point out the more recent Hexplorer approach by the Schaal laboratory which showed improved efficiency in current evaluation studies (see below) [34] In analogy with RESCUE-ESE, this approach relies on the computational comparison of hexamer frequencies in exonic and intronic sequences around weak and strong 5’ splice sites. Its advantage comes from counting each nucleotide’s probability of being part of an enhancer or a silencer from all the six hexamers overlapping it. Therefore, the program is able to better cover context dependence compared to methods based on individual predicted SRE motifs [34].

In parallel to analyzing existing genomic data, another way to define functional SREs is to use splicing reporter systems to test many different sequence combinations for enhancer or silencer properties. For example, Wang et al. adopted a systematic minigene analysis to test random decanucleotides for their ESS properties [33]. More specifically, they inserted random sequences into the middle exon of a three-exon minigene. The sequences that led to exon skipping of this otherwise constitutive exon were assigned as ESS. They can easily be searched using an online tool, FAS-ESS. Notably, one of the major drawbacks to this approach is that the discovered motifs might be functional only in the particular cellular and genomic (or minigene) context [2]. Later, Ke et al. used an analogous approach to assign enhancing or silencing properties to all hexanucleotide combinations (ESRseq scores) [32]. In order to make provision for the context dependence of the hexamer activities, these authors tested all the sequence motifs in five different exonic locations. Similar to the Hexplorer method, the ESRseq score for each hexamer was counted based on all the overlapping hexamers’ individual scores. Unfortunately, these scores are accessible only as supplementary tables to the article and no online program based on this approach has yet been made available to the public.

Recently, Xiong et al. [37] presented another approach based on splicing code modeling, which they used to design the SPANR tool (Splicing-based Analysis of Variants) [37]. The tool predicts the effects that a nucleotide variant exerts on cassette exon alternative splicing. This method extracts a huge number of DNA sequence features (*cis*-elements) from exon triplets (three exons and their intervening introns) and uses them to predict the percentage of middle exon inclusion. Owing to its non-linear nature, the model incorporates context dependent effects. In addition, the tool was trained on data from 16 different human tissues, which should enable it to make provision for some tissue specific features.

In addition to genomics and minigene systems, other possible approaches to identify splicing regulatory evidence have also been followed. In particular, Piva et al. have presented their database (called SpliceAid2) of human splicing factor expression data and RNA target motifs [38]. Here, users can get predictions on potential SREs based strictly on comparison with experimentally proved splicing regulators’ binding sites. The advantage of this approach is that it allows the identification of both the potential SRE element and the protein which may bind to it. Of course, the limitation is that we still largely ignore the binding site specificities of many RNA binding proteins. In addition, recent works on identifying RNA binding proteins in HeLa cells have uncovered a huge number of RNA binding proteins for which we completely ignore their potential to alter the splicing process [40]. 

Up until this moment, the best way to use these programs to obtain an accurate prediction of SRE elements in a given experimental system has been to use them in combination. For example, Raponi et al. adopted an integrated approach: they got the best predictions on mutation-induced exon skipping when they combined multiple individual SRE predictions together (see Table 2 for details) [41]. The resulting tools, EX-SKIP and HOT-SKIP, count the sum of ESEs and ESSs derived from individual predictions and then calculate the ESS/ESE ratio, either for the specific variant (in EX-SKIP) or for each possible single nucleotide substitution in a selected exon (in HOT-SKIP). An advantage of these tools is that the individual predictions are collectively shown on the results page, so the user does not have to approach each program individually. Such an advantage is also held by several online engines/web pages that include several individual prediction tools at one web location, e.g., Sroogle and Human Splicing Finder (Table 2) [26,42].

## 3. Efficiency of SRE Predictions

An important point about SRE predictors is their reliability in terms of concordance between predicted events and the real situation in cells, tissues and organisms. However, due to methodological constraints and lack of primary samples, most researchers often have had to rely only on in vitro results. Yet profound analyses showed rather good agreement between in vitro and in vivo splicing affection [48], suggesting that this drawback does not compromise substantially the validity of SRE predictions.

Despite the fact that developers of SRE-predictors mostly proved their functionality using independent in vitro analyses, additional studies often put the reliability of these tools in question. On one hand, SRE-prediction tools have been shown to recognize motifs with general splicing-regulatory properties. Particularly, the tools were demonstrated to statistically distinguish sequences with different propensity to activate cryptic splice sites or the splicing-affecting variants from harmless SNPs [30,49]. In parallel, Raponi et al. indicated several statistically significant correlations between SRE predictions and the level of *BRCA1* exon 6 inclusion [41]. On the other hand, difficulties arose when these predictors were tested for discerning individual splicing-affecting variants from harmless sequence changes. Many studies have therefore indicated SRE-predicting tools as less efficient, often inconclusive and difficult to interpret, possibly because most of these programs were not designed for this purpose [13,27,28,29,30,31]. For this reason, these programs have not been regarded as useful in clinical investigations [4].

Interestingly, several recent evaluations have pinpointed some promising achievements of newly developed algorithms (EX-SKIP, ESRseq scores and Hexplorer) to recognize SRE-affecting variants [13,15,19,45]. In particular, testing EX-SKIP with 29 variants found in five *CFTR* gene exons showed, on average, a 72.5% success rate in predicting the direction of exon inclusion change [45]. When assessing its capacity to distinguish variants capable of increasing exon skipping, the predictions suffered more from a lower sensitivity (71%) than specificity issues (75%). However, these numbers could be biased due to the higher number of mutations leading to exon skipping in the dataset compared to the silent variants and variants promoting exon inclusion. In another evaluation using 35 exonic variants in six different immunity-related genes, EX-SKIP showed reasonable sensitivity (75%) but poor specificity, possibly due to a low representation of splicing-affecting mutations in the testing data [19]. Likewise, Soukarieh et al. detected a similar sensitivity (75%) but again low specificity of EX-SKIP predictions (46%) [15].

For ESRseq scores, Di Giacomo et al. was the first study to independently show its promising potential in discerning splicing-affecting from non-affecting changes [13]. Using a tentative threshold for ESRseq score difference on 32 variants from *BRCA2* exon 7, they obtained no false negatives and just two false positive predictions on exon skipping induction. Later, Soukarieh et al. extended this analysis with four other sets of variants (from *MLH1* exon 10, *BRCA1* exon 6, *CFTR* exon 12 and *NF1* exon 37) including 154 individual point mutations in total [15]. The predictions on exon skipping variants in individual datasets showed sensitivity to be between 67% and 100% (weighed mean: 85%) and specificity between 66% and 94% (weighed mean: 83%). The high sensitivity of these predictions was later corroborated by Grodecká et al., although the specificity remained poorer [19].

Finally, the Hexplorer tool has been shown to perform comparably well with respect to the ESRseq scores. With the five extensive variant sets tested by Soukarieh et al., it provided a sensitivity between 57% and 100% (weighed mean: 79%) and specificity between 63% and 89% (weighed mean: 74%) [15]. In another study, this tool showed 100% sensitivity but again quite a poor specificity, possibly due to the chosen dataset [19].

In fact, all the above-described evaluations of EX-SKIP, ESRseq and Hexplorer have been done using data derived from minigene splicing analysis. In most cases, this analysis reliably mirrors the splicing effects, at least in terms of induction and the direction of a splicing defect [15,19,31,48]. 

Therefore, to extend these observations, we have decided to further evaluate these three predictors using 20 gene variants retrieved from the literature, for which the results on splicing affection were ascertained directly from the patients’ tissues. As shown in Table 3, even with this smaller set of mutations we have reached sensitivity and specificity in discerning skipping-inducing mutations comparable to the previous results [15,45], differing only in a somewhat higher specificity for the EX-SKIP tool compared to [15]. 

## 4. Exons Susceptibility to the Splicing Defects Due to SREs Changes

As mentioned in the introduction, some exons are being described as more susceptible to SRE disruptions than others. Typical signs for these susceptible exons are: (i) above- or below-average length (exon lengths below 50 nts and above 300 nts have been connected to lower splicing efficiency, probably due to affected recognition through the exon-definition process); (ii) weak splice sites; and (iii) alternative splicing [27,45,50,51]. However, these potentially important variables that affect the variants’ effects on splicing are not considered by most of the SRE-prediction algorithms. In addition, there are several other related factors that influence the basic level of exon recognition and might thus influence exons susceptibility to SRE-affecting variants, which might be more difficult to predict in silico. These are RNA secondary structure, nucleosome density and transcription rate [45]. Hand in hand with that, even distant regulatory elements in promoters and transcription enhancers have been shown to frequently influence alternative splicing. Besides impacting the basic level of exons’ recognition, this also represents another level of (hardly predictable) interindividual variability, as variations in these functional elements (e.g., SNPs or differences in methylation) are substantially associated with alternative splicing [52].

All these reasons may explain the discrepancies regarding results of SRE-predictor tools, that have been much more successful for some exons than for others. For example, compare the 57% vs. 83% sensitivity of Hexplorer predictions on variants in *MLH1* exon 10 and *NF1* exon 37, respectively, in Soukarieh et al. [15], or 87.5% vs. 50% success rate of EX-SKIP predictions on variants in *CFTR* exon 3 and exon 10, respectively, in Aissat et al. [45]. Hand in hand with that, while some predicted score differences showed significant correlations with the level of the induced exon skipping within individual exons, the overall extent of exon skipping between individual exons seems to be better predicted by the strength of splicing signals [15,45]. All these facts once again pinpoint the high context-dependence of the splicing regulation and the need for more complex prediction approaches to be developed.

## 5. Future Approaches at Evaluating the Effects of Splicing Disruption

The first issue, which still needs to be addressed by splicing research, is the prediction of the aberrant splicing pattern that emerges when splicing is affected. For obvious reasons, the particular outcome of a splicing-affecting mutation is crucial for diagnostic decisions. In general, the SRE-affecting mutations most usually increase or decrease inclusion of the exon they are located in [6,15,17,27,31]. However, they can also result in multiple exon skipping or activate a cryptic splice site [53,54,55]. In specific cases, intronic SRE mutations may lead to the activation of a pseudoexon (an intronic sequence that is neglected by the spliceosome under standard conditions) [56]. Once again, also in these cases the resulting splicing defects are extremely context-dependent.

Thus far, to the best of our knowledge, there were only a few pioneering attempts to use in silico prediction tools to assess whether a mutation will lead to exon skipping or to the cryptic splice site activation [57,58]. However, none of these approaches were directed at looking for potential SRE-affecting variants and it would be interesting if future research could specifically be targeted to this specific aim.

Finally, another issue that still needs to be addressed will be to predict the extent of aberrant splicing. This particular area is still in its infancy, with limited correlations between predicted variables and the level of exon skipping or inclusion [15,45]. Such correlations might be useful for prediction tool developers, but they are rather useless for the diagnosticians who have to cope with individual sequence changes. In particular, all these approaches often disregard the fact that for many genes, the level of exon inclusion is often tissue specific [6,27]. This problem will eventually have to be included in predictions, if they are ever going to be useful in diagnostics.

## 6. Clinical Significance of Splicing Aberrations

As outlined above, if we consider the huge complexity of causes and consequences of splicing aberrations, one can easily understand the challenges that a genetic diagnostician can meet when working with novel gene variants. As a result, extensive effort has been put into defining classification criteria for assessment of variants’ pathogenicity, as this issue markedly impacts patients’ clinical management. Some publications proposed criteria for overall pathogenicity assessment, either for disease-causing genes in general or for a specific set of genes (e.g., mismatch repair genes) [52,53]. Others aimed more specifically at coping with splicing-affecting mutations [54,55]. All these works based their classification on a five-tier scheme where the most probably pathogenic variants fall into the class 5 while those most probably benign are included in class 1. For clarity, we have summarized the classification criteria that apply to SRE-affecting variants in Table 4.

Regarding splicing alterations, variants leading to aberrant splicing of 100% transcripts, with clear effect on protein function (i.e., introducing PTC or in-frame deletion disrupting a functional domain) fall into class 5 (classified as pathogenic) [59,60]. According to some guidelines, aberrant splicing has to be shown on patient’s mRNA samples [59], while others allow this classification to also be based on in vitro assays when the results are supported by additional clinical evidence (family co-segregation, number of tumors, etc.) [60,61].

According to Thompson et al. [60], variants shown to abrogate mRNA splicing using in vitro assays only (without patient’s RNA-derived data) can fall into class 4 (likely pathogenic), if the additional clinical evidence does not allow class 5 ranking. On the other hand, after careful comparison of results obtained from patients’ RNA and the minigenes, van der Klift et al. proposed that minigene assay alone should be sufficient for class 4 of clinical classification, if a splicing affecting variant demonstrates complete aberrant and frameshifting effect [48]. Such reclassification would have important consequences for patients, since class 4 and class 5 variants are recommended to have the same clinical management. 

However, after analyzing data from several systemic splicing studies, we found that only a tiny minority of SRE-affecting mutations led to a complete splicing defect in minigenes (in particular, 5 of 136, i.e. 3.7%, variants increasing exon skipping showed 100% splicing aberration) [14,15,17,27,63]. From this point of view, the guidelines proposed by Richards et al. seem to be more pertinent for the SRE-affecting variants, since these always use a combination of several criteria to classify a variant and do not stick to 100% aberrant effect at the same time [61]. 

This pinpoints another difficulty that arises from the uncertainty in the minimal extent of aberrant splicing that can lead to a particular disease. In other words, the minimal amount of the residual full-length transcript sufficient to cover the biological function (in the relevant tissue) and prevent the disease development needs to be determined for individual conditions. Importantly, we cannot forget any possible adverse effects that the products of aberrantly spliced transcripts may exert on the wild type ones, e.g., dominant negativity in protein dimers [55,64].

At present, it follows that many variants fall into class 3—uncertain pathogenicity. Besides the mutations resulting in leaky aberrant splicing (variant allele produces full-length transcripts to some extent), this pertains, e.g., for exon-skipping mutations leading to in frame deletions not disrupting known functional domains or to upregulation of physiological alternative transcripts, at least some of which do not clearly disrupt protein function [59,60].

While the splice site related predictions are beginning to be included in classification guidelines, the SRE-related predictions stand aside [59,61]. In fact, despite the significant progress of these programs achieved in the past few years, they still await proper validation [4,15,17,48]. Currently, we can propose using the best performing tools (ESRseq, Hexplorer, and possibly EX-SKIP), ideally in combination with some a priori knowledge about the exons alternative splicing, as a rough preliminary filter to select the variants for further in vitro testing. Naturally, such a selection should be based on an overall estimation of each case (careful assessment of the patient’s condition, variant co-segregation, etc.). Recognition of the splicing affecting variants is beneficial not only for the diagnostics, but it may eventually lead to the development of effective RNA-targeting therapy. This issue has been addressed by several recent reviews [65,66,67,68].

## 7. Conclusions

In the past two decades, many prediction tools have assisted researchers in defining potential SREs. On the other hand, only the approaches that either consider a nucleotide change in its larger sequence environment or combine several individual prediction methods have been successful in accurately recognizing splicing-affecting SRE changes. Repeatedly, the best outcomes have been shown for ESRseq scores, followed by Hexplorer and EX-SKIP tools. What still prevents these tools from being firmly included in diagnostic settings is their somewhat variable reliability for different exons, resulting defect ambiguity, and the limited number of evaluating studies.

In general, the future aim for SRE predictors is to make maximal provision for the context dependence of splicing regulation to improve both the recognition of splicing-affecting variants and the assessment of the resulting defects. To this point, combining the SRE predictions with an estimate of exon susceptibility to be skipped or to activate nearby cryptic splice sites may be helpful. Other approaches could include data gained from functional (high throughput) SRE mapping or from CLIP-detected binding sites of splicing regulators, accompanied with the knowledge of splicing effects upon their knock down.

## Figures and Tables

**Table 1:** Selected individual SRE-prediction tools.

Prediction Tool	Principle	Website	Reference	Evaluation
ESE-finder	SELEX (in vitro selection of ligands)	http://krainer01.cshl.edu/cgi-bin/tools/ESE3/esefinder.cgi?process=home	[24]	[15,19,27,28,29,30,31]
ESRseq	testing of all possible k-mers for positive and negative splicing influences, based on QUEPASA method		[32]	[13,15,19]
FAS-ESS	analysis of random sequence silencing properties in the minigene settings	http://genes.mit.edu/fas-ess/	[33]	[30]
Hexplorer	statistical comparison of hexamer sequence motifs	http://nar.oxfordjournals.org/content/early/2014/08/21/nar.gku736/suppl/DC1	[34]	[15,19]
PESX	statistical comparison of octamer sequence motifs	http://cubio.biology.columbia.edu/pesx/pesx/	[35]	[19,27]
RESCUE-ESE	statistical comparison of hexamer sequence motifs	http://genes.mit.edu/burgelab/rescue-ese/	[36]	[19,27,30,31]
SPANR	splicing code, machine learning	http://tools.genes.toronto.edu/	[37]	[15]
SpliceAid2	database of in vitro proved splicing factors binding sites	www.introni.it/spliceaid.html	[38]	

**Table 2:** Selected tools and online engines combining multiple SRE-prediction tools.

Prediction Tool	Included Tools	Website	Reference	Evaluation
EX-SKIP	PESE and PESS [35], FAS-ESS [33], RESCUE-ESE [36], EIEs and IIEs [43], NI-ESE and NI-ESS [44]	http://ex-skip.img.cas.cz/	[41]	[13,15,19,31,45]
HOT-SKIP	PESE and PESS, FAS-ESS, RESCUE-ESE, EIEs and IIEs, NI-ESE and NI-ESS	http://hot-skip.img.cas.cz/	[41]	
Human Splicing Finder (HSF)	ESE-finder [24], RESCUE-ESE, PESE and PESS, EIEs and IIEs, FAS-ESS and ESS decamers [33], Exonic splicing regulatory sequences [5], HSF- specific matrices for Tra2-β, 9G8 and hnRNP A1 [26]	http://www.umd.be/HSF3/index.html	[26]	[15]
Sroogle	ESE-finder, RESCUE-ESE, FAS-ESS, PESE and PESS, other SRE predictions according to Voelker [46], Yeo [47], Goren [5]	http://sroogle.tau.ac.il/	[42]	

**Table 3:** Evaluation of ESRseq, Hexplorer and EX-SKIP predictors on patients’ RNA-based results.

Gene	cDNA Variant	Exon	Effect on Exon Skipping	ΔESRseq (−0.5)	Hexplorer: ΔHZ_EI_ (−0.5)	EX-SKIP: ESS/ESE mut/wt (1)
*BRCA1*	c.5123C > A	18	increased	−2.574	-10.85	1.05
	c.5434C > G	23	increased	0.558	−1.28	1.09
	c.5453A > G	23	increased	−2.176	−15.02	1.43
	c.5096G > A	18	none	−1.731	−0.22	0.93
	c.5116G > A	18	none	1.582	2.67	0.86
	c.5411T > A	23	none	0.66	4.33	0.83
*BRCA2*	c.231T > G	3	increased	1.65	4.76	0.97
	c.439C > T	5	increased	−2.69	−13.06	1.77
	c.7992T > A	18	increased	−1.11	0.00	1.00
	c.8257_8259delCTT	18	increased	−0.51	0.52	0.98
	c.9234C > T	24	increased	−1.24	−12.07	1.12
	c.223 > C	3	none	0.37	−11.19	0.97
	c.433_435delGTT	5	none	−0.23	6.46	0.51
	c.7994A > G	18	none	−1.21	0.24	1.02
	c.8182G > A	18	none	−1.65	0.00	0.98
	c.9216G > 1	24	none	−2.88	−10.94	1.04
*NF1*	c.557A > T	5	increased	−2.43	−12.21	1.25
	c.528T > A	5	none	1.17	0.70	0.90
*DMD*	c.5287C > T	37	increased	−0.70	−16.84	1.16
	c.5308A > T	37	none	−0.05	−2.85	1.05
True calls				70.0%	70.0%	70.0%
Sensitivity				80.0%	70.0%	70.0%
Specificity				60.0%	70.0%	70.0%

To further evaluate the three prediction tools, we have retrieved 20 gene variants detected in genes *BRCA1*, *BRCA2*, *NF1* and *DMD* (10 inducing aberrant splicing and 10 harmless at the level of splicing) from the literature. In all these cases, nonsense mediated decay was either prevented or not expected. For an easy comparison, we used the same thresholds as described in Soukarieh et al. (shown in table headings) [15], except from the original Hexplorer threshold which was not applicable to our data, so we used a threshold −0.5 instead. The true calls are shown in bold. ΔESRseq: score difference between predicted mutant and wild type ESRseq score. ΔHZ_EI_: score difference between predicted mutant and wild type HZ_EI_ score.

**Table 4:** Summary of clinical classification guidelines that apply to SRE-affecting variants.

Class		Observation	Reference
5: pathogenic	•	assay on mRNA from patients tissue samples	[59] ^1^ [60]
	AND	no wt transcript detected from variant allele	
	AND	aberrant transcripts introduce PTC or deletion disrupting functional domain	
		OR deletion disrupting protein conformation	only in [60]
	OR	damaging effect on the gene or gene product (extent not specified)	[61]
	AND	other lines of evidence supporting variant pathogenicity ^2^ (stronger than for class 4)	
	•	lab assays based on mRNA (e.g., minigenes)	[60,61]
	AND	variant-specific abrogated function (extent not specified)	
	AND	additional frequency/co-segregation/clinical data, additional molecular/mechanistic evidences from other sources, supporting variant pathogenicity (stronger than for class 4)	
4: probably pathogenic	•	assay on mRNA from patients tissue samples	[61]
	AND	damaging effect on the gene or gene product (extent not specified)	
	AND	other lines of evidence supporting variant pathogenicity (milder than for class 5)	
	•	lab assays based on mRNA (e.g., minigenes)	[60,61]
	AND	variant-specific abrogated function (extent not specified)	
	AND	additional frequency/co-segregation/clinical data, additional molecular/mechanistic evidences from other sources, supporting variant pathogenicity (milder than for class 5)	
	•	minigene assays	[48]
	AND	complete aberrant and frameshifting effect/ deletion of a functional domain effect	
3: uncertain pathogenicity		all variants that do not fall into other classes	[59]
	e.g.,	aberrant transcripts produce deletion not disrupting known functional domains	
	e.g.,	change in the level of alternative transcripts, at least some of which do not introduce PTC or protein-disrupting deletion	
	e.g.,	leaky aberrant splicing	
	e.g.,	contradictory benign and pathogenic criteria	[61]
2: likely not pathogenic	•	assay on mRNA from patients tissue samples	[59,60,61]
	AND	no associated mRNA aberration detected	
	AND	analysis including NMD inhibition	only [60]
	AND	other lines of evidence disproving variant pathogenicity	only [61]
	•	lab assays based on mRNA (e.g., minigenes)	[60,61]
	AND	variant-specific proficient function	
	AND	additional frequency/co-segregation/clinical data, additional molecular/mechanistic evidences from other sources, disproving variant pathogenicity (milder than for class 1)	
1: not pathogenic	•	lab assays based on mRNA (e.g., minigenes)	[60,61]
	AND	variant-specific proficient function	
	AND	additional frequency/co-segregation/clinical data, additional molecular/mechanistic evidences from other sources, disproving variant pathogenicity (stronger than for class 2)	

**^1^** Richards et al. combines multiple individual pathogenic and benign criteria to reach the classification [61]; ^2^ Walker et al. recommends predicted/expected SRE aberrations only for research testing [59]. • denotes a single set of criteria for each class. A variant has to meet all the criteria in at least one set to be included into the respective class.

The criteria listed in the table were extracted from several publications that were written, in fact, for somewhat differing purposes. Walker et al. published an evaluation and update of clinical classification guidelines previously designed by Spurdle et al. that were specifically aimed at potential splicing affection [59,62]. On the other hand, Thompson et al. and Richards et al. dealt with overall pathogenic potential of novel variants including direct effects on protein coding [60,61]. While Thompson et al. limited their criteria on mismatch repair genes [60], Richards et al. designed criteria for classification of all variants identified in genes that cause Mendelian disorders [61]. Finally, we have included one classification proposal derived from a publication that thoroughly evaluated the reliability of minigenes [48]. Of note, we did not include in the table the splicing-specific classification designed by Houdayer et al. [17], as this recommended a particular (specific) re-classification of class 3 variants (or variants of unknown significance) into three other classes (1S, 2S, 3S).

Concerning the in silico predictions, Richards et al. propose using them as a supportive criterion, if all the in silico programs tested agree on the prediction [61]. However, they do not directly mention predictions of SREs in their publication, describing only the splice site predictors. In comparison, Walker et al. allows usage of SRE predictors in general. If a variant causes a loss or creation of the same SRE predicted by at least two of three used programs, then these guidelines propose that it should be experimentally tested (as a research testing) [59]. Other guidelines mentioned in this table do not propose the usage of SRE predictions. Please note that only an effect on splicing is taken into account in this table. The effect of a variant on protein coding per se should always be considered as well.

## Abbreviations

CERES	Composite exonic regulatory element of splicing
circRNA	Circular RNA
CLIP	Cross-linking immunoprecipitation
ESE	Exonic splicing enhancer
ESS	Exonic splicing silencer
HSF	Human Splicing Finder
ISE	Intronic splicing enhancer
ISS	Intronic splicing silencer
PTC	Premature termination codon
RESCUE	Relative Enhancer and Silencer Classification by Unanimous Enrichment
SELEX	Functional systematic evolution of ligands by exponential enrichment
SNP	Single nucleotide polymorphism
SPANR	Splicing-based Analysis of Variants
SRE	Splicing regulatory element
